# Ultrasonography for lymph nodes metastasis identification in bitches with mammary neoplasms

**DOI:** 10.1038/s41598-018-34806-9

**Published:** 2018-12-07

**Authors:** Priscila Silva, Ricardo Andres Ramirez Uscategui, Marjury Cristina Maronezi, Beatriz Gasser, Letícia Pavan, Igor Renan Honorato Gatto, Vivian Tavares de Almeida, Wilter Ricardo Russiano Vicente, Marcus Antônio Rossi Feliciano

**Affiliations:** 1 Department of Clinic and Veterinary Surgery, São Paulo State University (Unesp), School of Agricultural and Veterinarian Sciences, Jaboticabal, Jaboticabal, Sao Paulo Brazil; 2 Institute of Agrarian Sciences, Federal University of the Jequitinhonha and Mucuri Valleys (UFVJM), Unaí, Minas Gerais Brazil; 3 Department of Preventive Veterinary Medicine and Animal Reproduction, São Paulo State University (Unesp), School of Agricultural and Veterinarian Sciences, Jaboticabal, Jaboticabal, Sao Paulo Brazil; 4 Department of Veterinary Radiology, Federal University of Recôncavo da Bahia (UFRB), Cruz das Almas, Bahia Brazil

## Abstract

The aim of this study was to evaluate and compare the diagnostic accuracy of B-mode, Doppler ultrasonography and Acoustic Radiation Force Impulse (ARFI) elastography in the identification of axillary and inguinal lymph nodes metastasis in bitches with mammary neoplasms. The axillary (n = 96) and inguinal (n = 100) lymph nodes of 100 bitches were evaluated using B-Mode, Colour Doppler and ARFI-elastography. After this evaluation, mastectomy and lymph nodes excision were performed and these structures were histologically classified as free, reactive or metastatic. Ultrasonographic parameters were compared by Chi-Square or ANOVA tests and if they are significant, discriminative power analysis according to histopathological classification was performed (ROC analysis). The ARFI-elastography shear wave velocity (SWV) enabled metastasis identification in inguinal (sensitivity 95% specificity 87%) and axillary lymph nodes (sensitivity 100% specificity 94%). While B-Mode ultrasound Short/Long axis ratio evaluation of inguinal and axillary lymph nodes only resulted in a sensitivity around of 71% and specificity of 55%. In conclusion, B-Mode ultrasonography may contribute to diagnosis of metastasis in axillary and inguinal lymph nodes of bitches affected by mammary neoplasm with limited accuracy, while SWV evaluation proved to be an excellent diagnosis tool, which allows differentiation between free, reactive and tumour metastatic lymph nodes.

## Introduction

The mammary neoplasms in bitches and women exhibit similar biological behavior^1–3^, its metastatic capacity can reach 50% and it is the main cause of mortality^4–6^. Metastasis occur mainly to axillary and inguinal lymph nodes in bitches^7^ and to axillary in women^8^, being an unfavourable prognosis factor for any of these patients^9,10^. Thus, the clinical evaluation of these structures becomes essential for adequate tumour staging, selection of therapeutic management, planning surgical margins and issuing a precise prognosis^10,11^. Clinical, radiologic, ultrasonographic and cytological examination may aid in the presumption of lymph nodes metastases, although they are not accurate in detection of micro metastasis and neoplastic cells clusters^12^.

Therefore, definitive diagnosis of lymph nodes metastasis is based on histopathological examination, which requires surgical excision^13^ a procedure with highly complications risk, mainly due to proximity of large vessels and nervous plexus to the axillary lymph node. These risks limit this technique application, even though it is advocated by the “Consensus for the diagnosis, prognosis and treatment of canine mammary neoplasms” as essential for patient diagnosis and prognosis^10,11,14^. The clinical and ultrasonographic examination of the regional lymph nodes are useful in the attempt to identify metastasis in a non-invasive and risk-free way. However, the few available analyses are considered limited because they use features such as increased size, central distribution of vascular flow and elevation of vascular indices as suggestive of metastasis without indicating the sensitivity and specificity, which is necessary to imply these techniques as diagnostic methods^13,15–17^.

With the advancement of ultrasonography, elastography has emerged as a method that allows a non-invasive evaluation of tissue elasticity and has become an efficient tool for identification of malignant mammary lesions in humans and animals^18,19^. Recently, clinical studies have evaluated the accuracy of this technique to identify metastasis in axillary lymph nodes of women with breast cancer and presented extremely promising results^20–22^. However, elastography studies were not found in canines.

Considering the requirement of non-invasive, accurate and risk-free identification of regional lymph nodes metastasis in bitches added to the promising results found by previous elastography studies in women, this study aims to evaluate and compare the accuracy of B-mode, Doppler and ARFI-elastography techniques for diagnosis of axillary and inguinal lymph nodes metastasis in bitches with mammary neoplasms.

## Results

A total of 100 bitches with 242 breast lesions (44 different neoplastic types) were included in this study. The Table 1 presents the site of the mammary glands bearing the neoplasms. Of these patients, 100 inguinal and 96 axillary lymph nodes were surgically collected and histologically analysed (four axillary lymph nodes were not collected due to impossibility of surgical identification). In this study, only one axillary lymph node was identified in the 96 bitches studied (all animals), two inguinal lymph nodes in 6 bitches and one inguinal lymph node in the remaining 94 bitches. The histopathological results of multiple inguinal lymph nodes were same in all 6 cases.

**Table 1:** Mammary glands bearing neoplasms.

Mammary gland (site)	Number of neoplasms
1^st^ Cranial thoracic	15
2^nd^ Cranial thoracic	29
3^rd^ Cranial abdominal	81
4^th^ Caudal Abdominal	85
5^th^ Inguinal	32
Total neoplasms	242

From 100 inguinal lymph nodes, 30% (30) were histopathological classified as free, 51% (51) as reactive, and 19% (19) as metastatic. Out of the 96 axillary lymph nodes, 35% (33) were classified as free, 56% (54) as reactive, and 9% (9) as metastatic. Excision of the axillary lymph nodes did not result in major postoperative complications, only 5 large animals presented transient subcutaneous oedema (seroma), probably due to the deeper location of this structure that warrants greater manipulation than in small and medium-sized animals.

Ultrasound evaluation was performed without difficulties, intercurrences or side effects. The qualitative and quantitative findings of inguinal lymph nodes evaluation are summarized in the Table 2, and those of the axillary lymph nodes in the Table 3.

**Table 2:** Qualitative variables in percentage of cases; and Mean ± SD of quantitative variables evaluated by different ultrasonography methods (B-mode, Doppler and ARFI-elastography) in inguinal lymph nodes of bitches with mammary neoplasms.

Parameter		Free	Reactive	Metastatic	P-value
**B-Mode ultrasonography**					
Shape	Elongated	80% (24)	31% (16)	22% (4)	0,6104
	Rounded	20% (6)	69% (35)	78% (15)	
Echotexture	Homogeneous	97% (29)	91% (46)	67% (13)	0,5017
	Heterogeneous	3% (1)	9% (5)	33% (6)	
	Mixed	0% (0)	0% (0)	0% (0)	
Echogenicity	Hypoechoic	97% (29)	98% (50)	89% (17)	0,2865
	Hyperechoic	3% (1)	2% (1)	0% (0)	
	Mixed	0% (0)	0% (0)	11% (2)	
Short/Long axis ratio		0,38 ± 0,10	0,37 ± 0,11	0,34 ± 0,10	0,4286
**Doppler ultrasonography**					
Vascularization	Present	50% (15)	58% (30)	60% (11)	0,1307
	Absent	50% (15)	42% (21)	40% (8)	
Localization	Peripheral	100% (30)	98% (50)	78% (15)	0,1537
	Central	0% (0)	2% (1)	22% (4)	
**ARFI-Elastography**					
Deformability	Hard	10% (3)^a^	15% (8)^a^	67% (13)^b^	0,0004*
	Soft	90% (27)	85% (43)	33% (6)	
Pattern	Homogeneous	97% (29)^a^	96% (49)^a^	44% (8)^b^	0,0009*
	Heterogeneous	3% (1)	4% (2)	56% (11)	
SWV (m/s)		1,91 ± 0,44^a^	2,29 ± 0,19^b^	2,99 ± 0,64^c^	0,0001*
Depth (cm)		1,05 ± 0,40	1,00 ± 0,38	1,10 ± 0,30	0,6075

cm: centimetres; s: seconds; m: meters; SD: standard deviation; *Considered statistically significant, where different letters indicate significance between the histopathological diagnoses (p < 0.05).

**Table 3:** Qualitative variables in percentage of cases; and Mean ± SD of quantitative variables evaluated by different ultrasonography methods (B-mode, Doppler and ARFI-elastography) in axillary lymph nodes of bitches with mammary lesions.

Parameter		Free	Reactive	Metastatic	P-value
**B-Mode ultrasonography**					
Shape	Elongated	45% (15)	31% (17)	22% (2)	0,1161
	Rounded	55% (18)	69% (37)	78% (7)	
Echotexture	Homogeneous	82% (27)	91% (49)	67% (6)	0,0949
	Heterogeneous	18% (6)	9% (5)	33% (3)	
	Mixed	0 (0)	0% (0)	0% (0)	
Echogenicity	Hypoechoic	97% (32)	98% (53)	89% (8)	0,1981
	Hyperechoic	3% (1)	2% (1)	0% (0)	
	Mixed	0% (0)	0% (0)	11% (1)	
Short/Long axis ratio		0,47 ± 0,13	0,49 ± 0,12	0,53 ± 0,16	0,4724
**Doppler ultrasonography**					
Vascularization	Present	10% (3)	28% (15)	22% (2)	0,3154
	Absent	90% (30)	72% (39)	78% (7)	
Localization	Peripheral	91% (30)	98% (53)	78% (7)	0,3858
	Central	9% (3)	2% (1)	22% (2)	
**ARFI-Elastography**					
Deformability	Hard	6% (2)^a^	15% (8)^b^	67% (6)^b^	0,0005*
	Soft	94% (31)	85% (46)	33% (3)	
Pattern (%)	Homogeneous	100% (33)^a^	96% (52)^a^	44% (4)^b^	0,0001*
	Heterogeneous	0% (0)	4% (2)	56% (5)	
SWV (m/s)		1,87 ± 0,27^a^	2,30 ± 0,35^b^	3,02 ± 0,50^c^	0,0001*
Depth (cm)		1,19 ± 0,36	1,34 ± 0,39	1,37 ± 0,52	0,1989

cm: centimetres; s: seconds; m: meters; SD: standard deviation; *Considered statistically significant, where different letters indicate significance between the histopathological diagnoses (p < 0.05).

**Figure 1:**
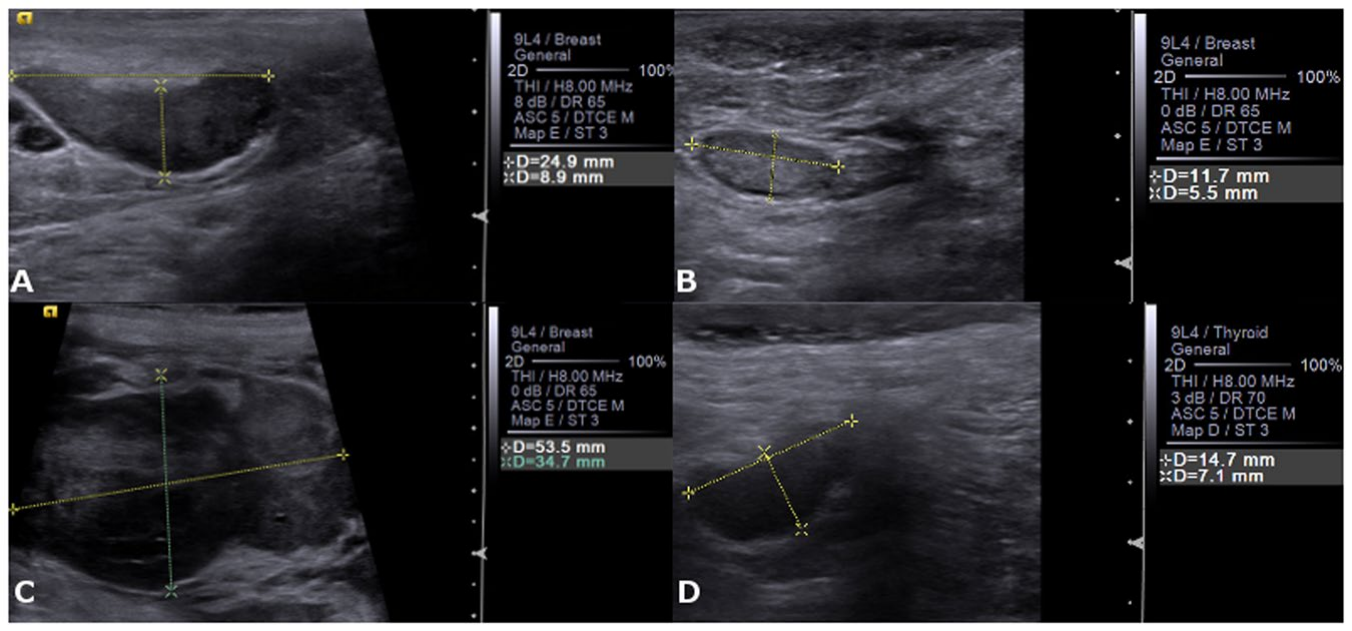
B-Mode ultrasound images of a canine loco regional lymph nodes. (**A**) inguinal lymph node with metastasis note the elongated shape. (**B**) normal inguinal lymph node. (**C**) axillary lymph node with metastasis note the rounded shape. (**D**) normal axillary lymph node.

**Figure 2:**
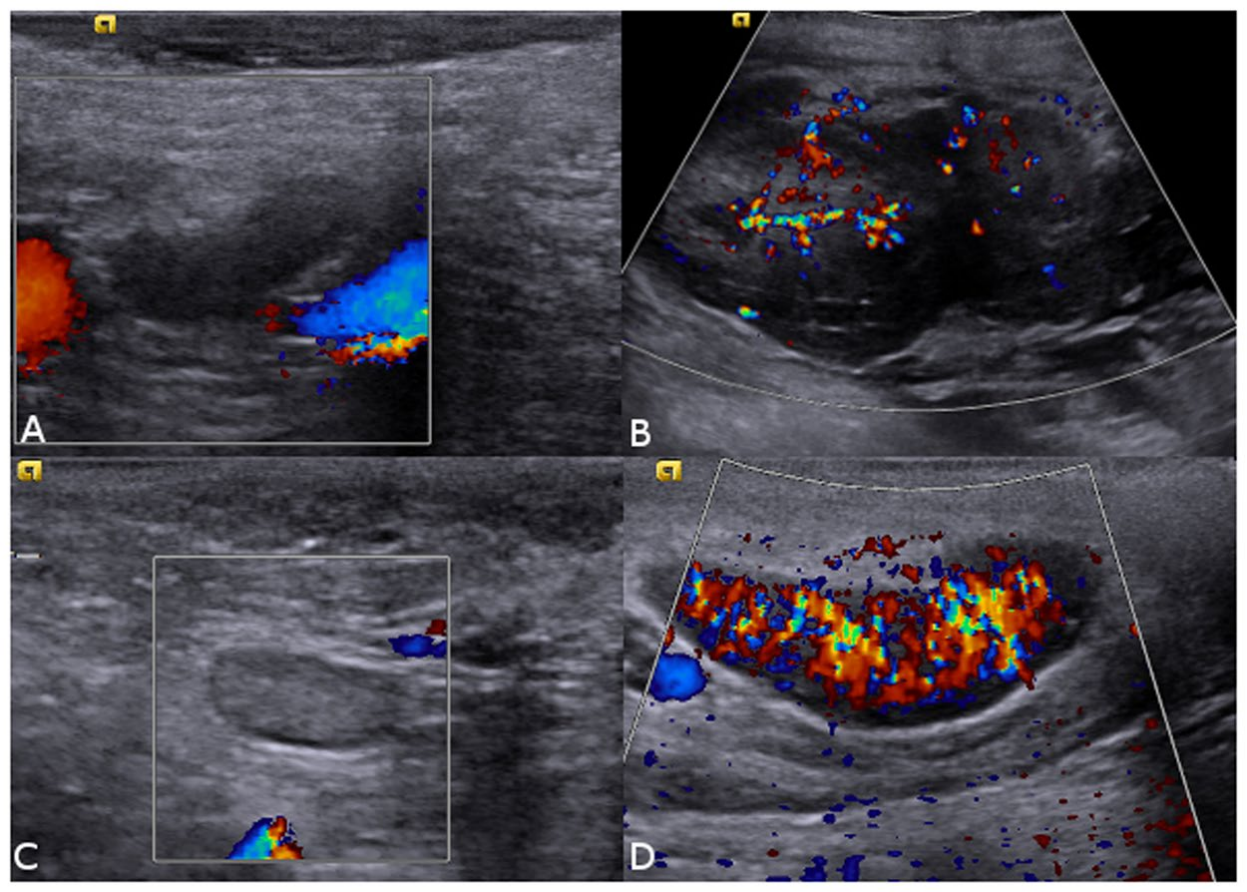
Colour Doppler ultrasound images of canine loco regional lymph nodes. (**A**) Normal axillary lymph node with absence of vascularization. (**B**) Axillary lymph node with metastasis and presence of neovascularization, (**C**) normal inguinal lymph node with absence of vascularization and (**D**) inguinal lymph node with metastasis and presence of neovascularization.

The B-Mode and Doppler ultrasound parameters were similar (P > 0.05) between the histopathological classification (Figs 1 and 2).

**Figure 3:**
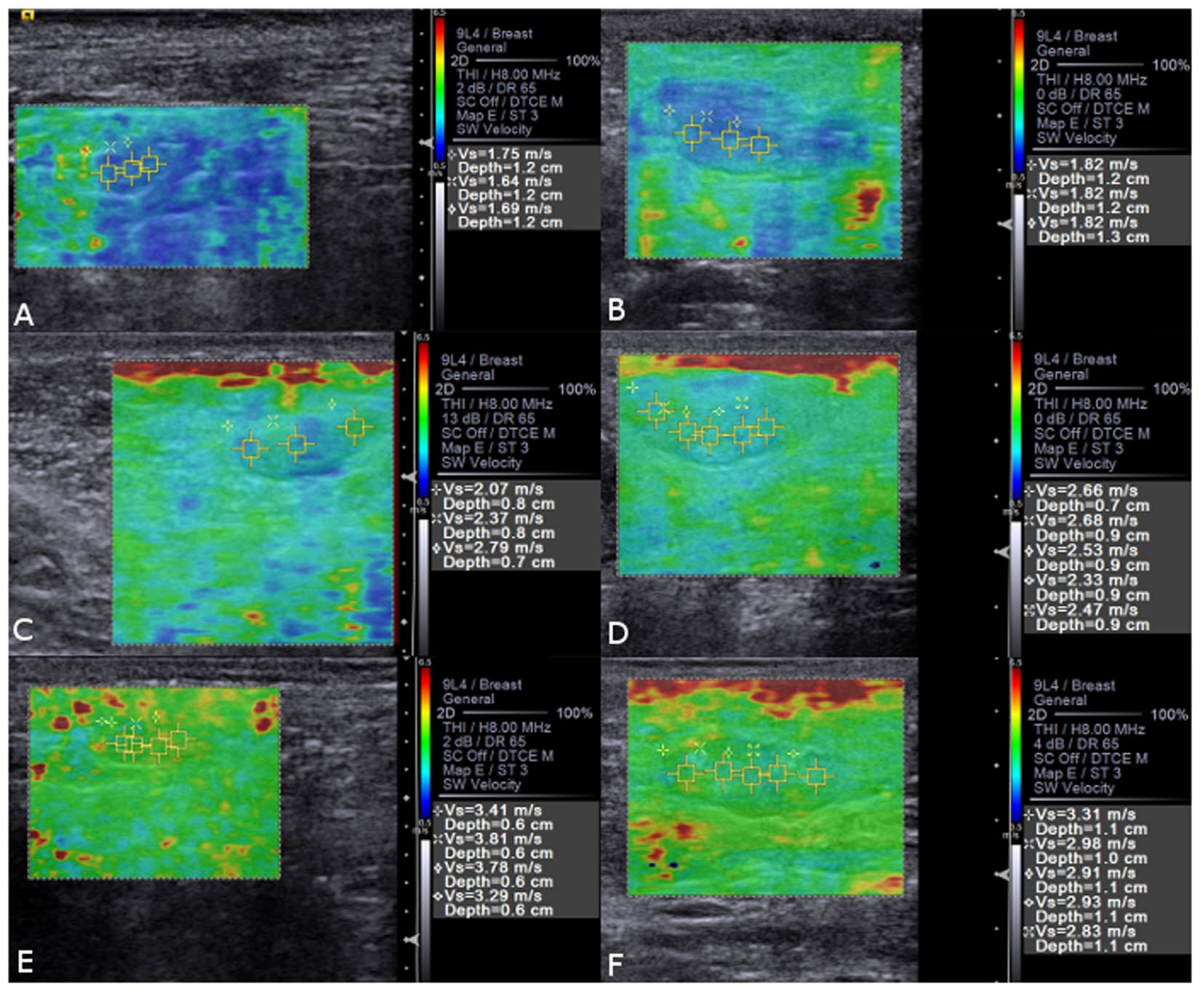
Qualitative and quantitative ARFI-elastography images of (**A**) normal axillary lymph node, (**B**) normal inguinal lymph node with homogeneous pattern and SWV in m/s. (**C**) Reactive axillary lymph node, (**D**) reactive inguinal lymph node with heterogeneous pattern and SWV in m/s. (**E**) Axillary lymph node with metastasis, (**F**) inguinal lymph node with metastasis with heterogeneous pattern and SWV in m/s.

At the ARFI-elastography, the qualitative evaluation revealed that lymph nodes with metastasis presented less deformability (P < 0.01) than normal lymph nodes. In the quantitative elastographic evaluation, SWV was greater (P < 0.01) in lymph nodes with metastasis than in reactive, and in these greater (P < 0.01) than in normal (Fig. 3).

**Figure 4:**
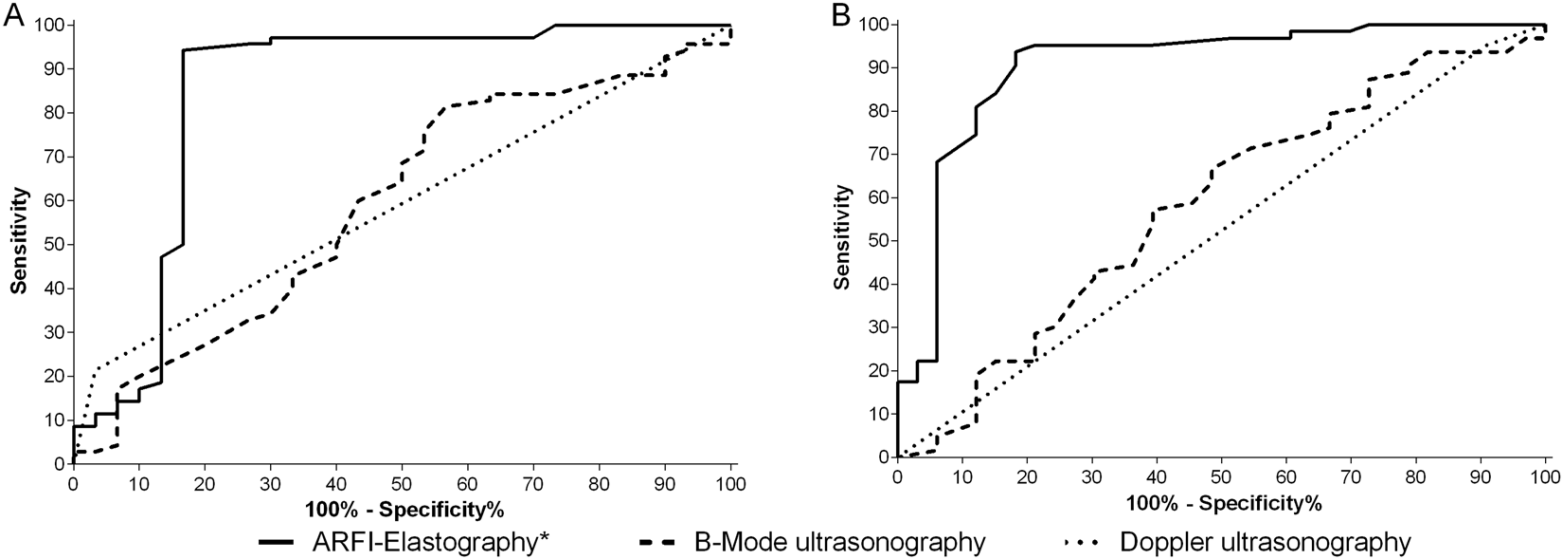
ROC (Receiving Operating Characteristic) curves that compare the predictive sensitivity % and 100% – specificity % of different ultrasound methods for determination of metastasis in (**A**) inguinal and (**B**) axillary lymph nodes of bitches affected by mammary neoplasms using the histopathological classification as reference. * indicate significant difference (P < 0.01).

The results of the discriminative power analysis for identification of inguinal and axillary lymph nodes metastatic, reactive or altered (metastatic + reactive) are shown in the Table 4, and the graphical representation of the comparative study of the ROC analysis, which allowed the identification of the lymph nodes SWV as the most applicable variable for distinguish between metastatic, reactive or altered (P < 0.01) is presented in Fig. 4.

**Table 4:** Diagnostic performance variables (%) of different ultrasound methods to predict metastasis, reactivity or alterations (metastasis + reactivity) in inguinal and axillary lymph nodes of bitches with mammary neoplasms.

Parameter	Diagnosis	Cut-off point	Sens%	Spec%	Accur%	AUC%
**Inguinal lymph nodes**						
Short/Long axis ratio	Altered	<0,41	76	47	67	59
	Metastatic	<0,38	63	41	45	56
Vascularization	Altered	Peripheral	22	97	44	59
	Metastatic	Peripheral	26	86	75	55
SWV (m/s)	Reactive	>2,1	92	83	89	82
	Metastatic	>2,5	95	87	90	92
	Altered	>2,1	94	83	90	85
Deformability	Metastatic	Hard	53	83	58	87
Pattern	Metastatic	Heterog	32	94	46	82
**Axillary lymph nodes**						
Short/Long axis ratio	Altered	>0,45	67	52	62	58
	Metastatic	>0,51	78	62	64	67
Vascularization	Altered	Peripheral	5	90	34	52
	Metastatic	Peripheral	18	95	86	60
SWV (m/s)	Reactive	>2,0	93	81	89	89
	Metastatic	>2,4	100	94	90	99
	Altered	>2,0	94	81	90	91
Deformability	Metastatic	Hard	67	80	86	69
Pattern	Metastatic	Heterog	56	98	94	86

CV: Cohort Value; Sens: Sensitivity; Spec: Specificity; Accur: Accuracy; AUC: area under curve; SWV: Share Wave Velocity; cm: centimetres; s: seconds; m: meters; Heterog: Heterogeneous.

## Discussion

B-mode ultrasound Short/Long axis ratio evaluation of axillary and inguinal lymph nodes in bitches with mammary neoplasms allowed identifying metastasis with moderate accuracy. The evaluation of the SWV by ARFI-elastography proved to be a technique with exceptional accuracy in these structures, while the vascular evaluation by Colour Doppler did not present any parameter that would allow determining metastasis in these organs.

The SWV was significantly higher in metastatic and reactive tissues compared to free tissues, with a general discriminative accuracy to identify altered lymphatic tissues around 90%, proving to be a suitable, accurate and non-invasive method for both altered differentiation and malignancy diagnosis in axillary and inguinal lymph nodes of bitches with mammary neoplasms. This increase in metastatic lymph nodes stiffness corroborates the results reported in women by Tamaki *et al*.^21^, who described a SWV cohort value >1.44 m/s, with sensitivity of 83% and specificity of 70% for metastasis detection in axillary lymph nodes. Our results indicate a great SWV cohort value >2.5 m/s, with great sensitivity of 95% and specificity of 87%, for axillary lymph nodes metastasis identification.

The findings obtained in the qualitative ARFI- elastography evaluation were similar to those described by Choi *et al*.^23^ in women metastatic lymph nodes, who described greater rigidity in these structures (areas with reddish tones) than in reactive ones (areas with greenish tones) and free ones (areas with bluish tones). Our results also corroborate with Seiler *et al*.^24^, that observed that malignant tissues present higher scores of tissue stiffness through compression elastography and computational analysis of the images in different lymph nodes of 51 canine and feline patients. Regarding the qualitative evaluation of deformation and homogeneity, no differences were observed among the tissues studied, since these may exhibit similar parenchymal variations^10^.

The qualitative and quantitative increase in tissue stiffness observed in reactive and metastatic lymph nodes is probably due to: abnormal cell proliferation, differentiation in areas with micro calcification and abnormal tissues deposition in the malignant tissue stroma^17,25^.

It is important to emphasize that results obtained with ARFI-elastography exam of sentinel lymph nodes can guide the adequate therapeutic management, tumour staging and prognosis formulation in patients affected by mammary neoplasms. It helps to determine the necessity of the axillary lymph node excision and, consequently, the extension of the surgical procedure, which may limit postoperative complications, common in this procedure^21,26^. Thus, it is expected that clinical application of this technique may not only reduce complications rates by saving the patient from unnecessary trauma when the lymph node is not affected, but also to adjust therapy and prognosis in patients with metastasis^21^.

On the other hand, the B-mode evaluation allowed determination that Short/Long axis ratio of inguinal and axillary lymph nodes were decreased and increased respectively when these tissues had metastasis, with a general discriminative accuracy around 60% for identification of altered lymph nodes. This result correlates with Choi *et al*.^27^ and Muramoto *et al*.^25^, that evaluated axillary lymph nodes of women and inguinal lymph nodes of bitches with mammary neoplasms, respectively. According to Chammas *et al*.^28^, inflamed lymphoid structures tend to increase their size proportionally in all planes whereas neoplastic lymph nodes grow disproportionately, losing their original shape, like that observed in the present study.

The lymph nodes short/long axis ratio evaluation demonstrate a moderated discriminative power to identify alterations studied in the regional lymph nodes, according to studies carried out in women by Vassallo *et al*.^29^ and bitches by Nyman *et al*.^15^ and Muramoto *et al*.^25^. These authors verified that this ratio increases in tissues with metastasis, while Muramoto *et al*.^25^ commented that no reference standards can be established for this relation due to the great variability of lymph nodes studied (high standard deviations). These controversial observations highlight the importance of this study regarding the use of modern sonographic techniques trying to improve the accuracy of metastasis diagnosis in sentinel lymph nodes.

The B-mode qualitative parameters (echogenicity and echotexture) did not demonstrate effectiveness to differentiate lymphoid tissue with metastasis nor reactive, corroborating with the previous reports^15,17,25^. The presence of necrotic, liquefaction or haemorrhagic areas, or areas that intersect normal and with tumour alterations tissues, micro calcifications and gross calcifications^13,28^ promote a tissue variability in the lymph nodes, which results in visualization of heterogeneous echotexture in all tissues evaluated^17,25^. Regarding echogenicity, variations can be observed in tissues with or without metastasis in women, which establish relation between reduction of echogenicity and increase in cellularity, observed in lesions as basic as hyperplasia^30^.

An unexpected result was that the vascularization detection and classification by colour Doppler did not allowed the differentiation of altered lymphoid tissues. This result may be correlated with the fact that variations in the vascularization of neoplastic and non-neoplastic tissues are due to inflammatory factors, observed in sentinel tissues with any inflammatory reaction which promotes detectable fluxes in normal and reactive lymph nodes and even less evident vascularization in ischemic foci derivate from metastatic lymph nodes necrosis^17,25^.

In conclusion, from the results of this study, B-mode ultrasonography can contribute to the diagnosis of metastasis in the axillary and inguinal lymph nodes of bitches affected by mammary neoplasms with limited accuracy. In contrast, the ARFI-elastography evaluation of SWV of these lymph nodes proved to be an excellent diagnostic technique, with high accuracy to differentiate reactive, metastatic or altered lymph nodes. It is recommended the inclusion of ARFI-elastography in the evaluation of the regional lymph nodes of oncologic patients because it is a rapid, non-invasive, complication-free, high sensitivity, specificity and accuracy technique capable of assist in the clinical/surgical management of patients affected by neoplasms.

## Methods

This study followed the recommendations of the Brazilian National Council for the Control of Animal Experimentation (CONCEA). Prospective clinical study, approved by the Ethics Committee in the Use of Animals of the São Paulo State University (Unesp), School of Agricultural and Veterinarian Sciences, Jaboticabal, São Paulo, Brazil (protocol n° 9.950/16), developed between March 2016 and August 2017. All bitches attend due to the presence of mammary lesions at the Institutional Veterinary Hospital, during this period were included after approval by free and informed consent by their tutors.

Single experienced sonographer (5 years) examined the axillary and inguinal lymph nodes with a 9.0 MHz linear transducer and Acuson S2000® equipment (Siemens, Munich, Germany), by the sonographic methods: B-mode, colour Doppler and ARFI-elastography.

At B-mode, the size (length and height) of the lymph nodes was measurement to calculate the short/long axis ratio, the shape was classified subjectively as elongated when one side was evidently larger than the other or rounded when the sides were apparently similar, the contours as regular or irregular, the echotexture as homogeneous or heterogeneous and the echogenicity classified according to adjacent tissues as hypoechoic, hyperechoic or mixed.

By Colour Doppler was studied the presence of vascularization and classified its localization as peripheral (apparently normal) or central (apparently neovascularization) using these image parameters: wall filter 3 Hz; pulse repetition 1099 Hz; gain 0db; peak hold 1 sec; colour sensitivity 4; persistence 2 level; mechanical index 0.9; and thermal index 1.8. Using a region of interest (ROI) that includes the lymph node parenchyma and at least 30% of adjacent tissue.

The ARFI-elastography was performed using the Virtual Touch Tissue Imaging Quantification (VTIQ) software^18^. This assembles a colour chart map known as elastogram, which assesses the qualitative deformability characteristic, where bluish tones correspond to deformable tissues (soft) and reddish to non-deformable tissues (hard). The elastogram was also classified as homogeneous when the image presented the same tone throughout the lymph node or as heterogeneous when presented different shades. Immediately thereafter, for quantitative evaluation, at least three ROI were selected by placing the 25 mm^2^ pre-defined calliper over the elastogram trying to cover the largest area of lymph nodes and include peripheral and central regions. The ultrasound software automatically provides the shear wave velocity (SWV m/s), of each of these ROI and the mean SWV was calculate for each lymph node.

After ultrasound examination, the patients were submitted to unilateral radical mastectomy and ipsilateral axillary and inguinal lymph nodes excision. To confirm that the axillar lymph node extracted was the same one examined, some illegibility criteria were adopted: (1) the ultrasound exam and the excision were performed on the same day; (2) the patent blue injection was made immediately previous to surgical approach to confirm the detection and excision of the sentinel lymph node; and (3) the measurements and morphological aspects obtained in the ultrasound were compared with the dimensions and macroscopic morphological aspects at surgery excision. And in relation to the inguinal lymph node, it is noteworthy that the entire mammary chain and inguinal lymph nodes are removed and sent as a block for histopathological analysis, so if there were more than one inguinal lymph node it would be confirmed by the pathologist. The lymph nodes and mammary tissues were preserved in 10% formalin, routinely processed and examined under a light microscope by a single experienced pathologist (15 years) who determines the neoplasms diagnosis and classifies the lymph nodes as free (absence of histopathological changes), reactive (inflammatory alterations) or metastatic (micro metastasis or neoplastic cells clusters) following the recommendation of Cassali *et al*.^10,21^.

Statistical analysis was performed using the software R, version 3.3.0 (R® foundation for statistical computing, Austria). Qualitative variables were compared between histopathological classifications by Chi-square test, quantitative variables by ANOVA test and differences were considered significant when P-value <0.05. For ultrasonography parameters that showed significance, the cut-off point, sensitivity, specificity, accuracy, and area under curve (AUC) were calculated using histopathological classifications as a reference for receiver-operating characteristic curve (ROC) analysis in a logistic regression model aimed at assessing and comparing the diagnostic performance of each technique.
